# Both Wnt signaling and epidermal stem cell-derived extracellular vesicles are involved in epidermal cell growth

**DOI:** 10.1186/s13287-020-01933-y

**Published:** 2020-09-23

**Authors:** Ling Leng, Jie Ma, Luye Lv, Wenjuan Wang, Dunqin Gao, Yunping Zhu, Zhihong Wu

**Affiliations:** 1 Stem Cell and Regenerative Medicine Lab, Department of Medical Science Research Center, Translational Medicine Center, Peking Union Medical College Hospital, Peking Union Medical College and Chinese Academy of Medical Sciences, Beijing, China; 2 State Key Laboratory of Proteomics, Beijing Proteome Research Center, National Center for Protein Sciences (Beijing), Beijing Institute of Life Omics, Beijing, China; 3 Institute of NBC Defense, Beijing, China; 4 Department of Dermatology, Chinese PLA General Hospital, Beijing, China; 5 Basic Medical School, Anhui Medical University, Hefei, Anhui China

**Keywords:** Epidermal stem cell, Extracellular vesicle, Organoids, Proteomics, Epidermal development, Extracellular matrix

## Abstract

Millions suffer from skin diseases. Functional interfollicular epidermal stem cells are needed in skin therapy or drug screening in vitro. We obtained functional interfollicular epidermal stem cells with intact stemness and cell junctions by treating them with Wnt3a. Moreover, epidermal stem cell-derived extracellular vesicles were useful in epidermal cell growth. Finally, functional epidermal 3D organoids with polarity were cultured using Wnt3a and the supernatant derived from interfollicular epidermal stem cells and fresh medium in a 1:1 ratio. These results provide novel directions for the improvement of skin organoids and their potential in clinical application.

## Background

In mammals, several distinct cell populations of the skin comprise three basic layers—epidermal layer, dermal layer, and hypodermis or subcutaneous fat layer. The epidermis is the outermost layer, composed of stratified cell layers maintained by keratinocytes, including both stem cells and the mature cells in abundance. The basal layer of the epidermis has undifferentiated proliferative progenitor cells expressing keratins, including keratin 5 (K5) and keratin 14 (K14) [1]. These progenitors not only replenish the basal layer through self-renewal, but also progressively migrate upwards through the epidermis, differentiating to form mature keratinocytes expressing keratin 1 (K1), keratin 10 (K10), and involucrin, and finally the outer layers of the terminally differentiated, dead stratum corneum cells [2]. Skin homeostasis needs two types of epidermal stem cells (EpSCs) including interfollicular epidermal stem cells and hair follicle stem cells. After injury, the interfollicular epidermal stem cells replicate and differentiate to form the mature epidermis and regenerate hair follicles. Wnt signaling pathways are involved in the maintenance of both the two types of stem cells, and play an essential role during skin development [2, 3]. Besides, the Wnt signaling pathways are important during wound healing [4] and regulate epidermal cell proliferation of skin EpSCs [5]. Canonical Wnt proteins result in the accumulation of unphosphorylated β-catenin protein stability by inhibiting GSK3β kinase [6], and β-catenin activates as a nuclear cofactor for the LEF1/TCF family of DNA-binding proteins to activate canonical Wnt down-stream pathways [7].

Various junctional and cytoskeletal proteins, including cytoskeletal building blocks and associated cell-matrix proteins or intercellular junctions, control the regenerative capacity of the epidermis in maintaining the EpSCs, which is useful for epidermal morphogenesis and growth [8]. For example, interfollicular epidermal stem cells adhere on a thin layer of specialized extracellular matrix (ECM) proteins between the epidermal and dermal layers called the basement membrane (BM). The BM is composed of type IV and VII collagen, laminin, perlecan, nidogens, growth factors, and other ECM proteins [9]. ECM proteins in the BM connect the basal stem cells through various anchoring complexes, which coordinate the actin and microtubule network and establish cell polarity, so that the basal stem cells (EpSCs) differentiate and proliferate [2]. The major receptors of ECM proteins which interact with EpSCs are integrins [1, 2]. Integrin subunit alpha 3 beta 1 (α3β1) and alpha 6 beta 4 (α6β4) are the major epidermal integrins that bind the ligand laminin-5 [2]. The downregulation of integrins reduces proliferation in the basal epidermal layers [10, 11]. Desmosomes are clustered transmembrane cadherins called desmogleins (DSGs) and desmocollins (DSCs) that bind to plakoglobin (PG) and plakophilins (PKPs) to form desmosomal complexes. Desmosomes directly link intercellular junctions and intermediate filament to regulate cellular growth and metabolism and support the cytoarchitecture of the epidermis [12, 13].

Millions suffer from skin diseases caused due to mutations or thermal and pressure injuries, such as epidermolysis bullosa (DB) or chronic diabetic ulcers [14]. With the development of the stem cell industry, functional seed cells are needed to treat patients with skin diseases or for drug screening. In addition, stem cell-derived extracellular vesicles can mediate cell-cell communication through cell-specific mRNA or proteins. Stem cell-derived, cell-specific extracellular vesicles can regulate biological function and enhance tissue regeneration [15–18]. However, the role of EpSC-derived extracellular vesicles on EpSCs is unclear.

In this study, we established both 2D and 3D EpSC culture system using Wnt3a protein. In addition, we first described the proteomic characteristics of EpSC-derived extracellular vesicles and their important roles on epidermal development that may benefit clinical application or drug discovery in the future.

## Materials and methods

### Human EpSC culture

Human skin tissue samples were obtained from Chinese PLA General Hospital, and ethical approval and informed consent were obtained. The interfollicular epidermal stem cells were isolated as described [19, 20] with some modifications. Briefly, subcutaneous tissues and fat were removed and the tissues were digested with Dispase II (2.5 mg/mL, Cat #17150041, Gibco) at 37 °C for 2 h to obtain the epidermal sheets. Thereafter, the samples were digested with pre-warmed Trypsin/EDTA (0.25%, Cat #25200072, Gibco) at 37 °C for 10 min, centrifuged at 1200×*g* for 3 min, and washed three times with PBS at 4 °C. Subsequently, the cells were seeded onto 6-well plates, pre-coated with Matrigel (Cat #354230, Corning). Advanced DMEM/F12 (Cat #12634, Invitrogen) medium containing NEAA (1%, Cat #11140, Gibco), B-27 (2%, Cat #17504, GIBCO), GlutaMAX (1%, Cat #35050, Gibco), HEPES (1%, Cat #15630, Gibco), N-Ace (1 mM, Cat #A9165, Sigma), hEGF (50 ng/mL, Cat #236-EG, R&D), A83-01 (2 μM, Cat #SML0788, Sigma), fosklin (10 μM, Cat #S2449, Selleck), and penicillin/streptomycin (100 U/L, Cat #15140163) with or without Wnt3a (50 ng/mL, Cat #5036-WN R&D) was prepared. The cells were incubated overnight and filmed using the Real-Time Cell History Recorder (JuLi stage, NanoEnTek Inc., Korea) inside the incubator. Images were taken every day using the bright channels. EpSCs (treated with or without Wnt3a or XAV939) were cultured in 96-well plates and counted daily for 5 days to perform the proliferation curve.

### 3D organoid culture

For 3D cultures, the cells obtained from the epidermis were cultured on ultra-low attachment culture dishes, in advanced DMEM/F12 medium with sodium hyaluronate (0.2%, Cat #H3506, Sigma) and Wnt3a for 5 days. The culture medium was partially replaced every alternate day, and XAV939 (1 μM, Cat #S1180, Selleck) was added, starting from day 5. The organoids were harvested after 5 days of culture for the next experiment.

### Immunofluorescence

Cells were fixed in 4% formaldehyde for 20 min and washed in PBS. Thereafter, the samples were treated with 0.25% Triton X-100 for 20 min, blocked in 10% serum for 1 h at 25 °C, and incubated with primary antibodies, such as COL17A1 (Cat #ab184996, Abcam), K14 (Cat #ab181595, Abcam), K10 (Cat #ab9026, Abcam), PLEC (Cat #sc-33,649, Proteintech), ITGB1 (Cat #ab170874, Abcam), ITGA6 (Cat #ab20142, Abcam), DSC2 (Cat #60239-1-Ig, Proteintech), DSG1 (Cat # 24587-1-AP, Proteintech), β-catenin (Cat #8480S, Cell Signaling), P63 (Cat #ab735, Abcam), CLDN1 (Cat #13050-1-AP, Proteintech), and Ki67 (Cat #ab15580, Abcam) overnight at 4 °C. Thereafter, the cells were incubated with secondary antibodies for 1 h at 25 °C, counterstained with DAPI, and the samples were sealed with Fluoro-Gel for Photography. The pictures were taken at × 20 or × 40 magnification and analyzed using Volocity Demo (× 64).

### qRT-PCR analysis

Total RNA was isolated using the RNeasy Mini Kit (Qiangen). Thereafter, the cDNA was synthesized using reverse transcriptase (ReverTra Ace® qPCR RT Master Mix, Toyobo), according to the manufacturer’s instructions. qRT-PCR was performed using the SYBR Green master mix (TOYOBO) on the Bio-Rad iQ5 Real-Time PCR detection system. Data were collected using the Bio-Rad CFX Manager software. Gene expression within a sample was normalized to GAPDH expression by the 2−ΔΔCt method. The primer sequences for PCR are available in Supplementary Table S1.

### Extracellular vesicle isolation and identification

The extracellular vesicles were isolated from the human epithelial stem cell (EpSC) culture supernatant using a differential centrifugation protocol [21, 22] with minor modifications. Briefly, the cells were grown in 10–15 cm^2^ flasks under normal culture conditions in media supplemented with 10–15% fetal bovine serum until they reached a confluency of 70–80%. Thereafter, the cells were cultured for > 48 h in media supplemented with extracellular vesicle-free FBS (A27208, Gibco, FBS was depleted of bovine extracellular vesicles by ultracentrifugation at 110,000 *g* for 180 min). The culture medium was collected and centrifuged at 300 *g* at 4 °C for 10 min and 2000 *g* at 4 °C for 20 min to remove dead cells. The supernatants were further centrifuged at 10,000 *g* for 30 min at 4 °C to eliminate contaminating cellular debris. The extracellular vesicles were pelleted from the final supernatants by ultracentrifugation for 70 min at 4 °C and 110,000 *g* (Beckman SW32Ti rotor) using an SW32 Ti swinging bucket rotor (Beckman Coulter, Fullerton, CA). The pellets were washed in PBS to eliminate contaminating proteins. Another round of centrifugation was performed at high speed and the supernatants were discarded. The extracellular vesicles samples were used for transmission electron microscopy (TEM) and western blot analysis.

### TEM analysis

The samples were washed in PBS and fixed overnight in 0.1% formalin and for 2 h in 2.5% glutaraldehyde. Thereafter, the samples were washed in PBS, dehydrated in ascending concentrations of ethanol, and air-dried. The ultrastructure of artificial skins was gold sputtered and mounted for imaging on a transmission electron microscope.

### Western blot analysis

The extracellular vesicles were collected and homogenized on ice in RIPA buffer, supplemented with a protease inhibitor cocktail (04693116001, Roche). For immunoblotting, equal amounts (20 μg per lane) of extracellular vesicle lysates were separated on a 10% SDS polyacrylamide gel. The samples were blocked with 5% skim milk for 2 h and incubated with anti-CD9 (60232-1-Ig, Proteintech, 1:1000 dilution), K1 (Cat #ab93652, Abcam, 1:1000 dilution), K14 (Cat #ab181595, Abcam, 1:20,000 dilution), ITGB1 (Cat #ab170874, Abcam, 1:1000 dilution), ITGA6 (27189-1-AP, Proteintech, 1:1000 dilution), ERK1 (16443-1-AP, Proteintech, 1:1000 dilution), ECAD (Cat #ab76055, Abcam, 1:1000 dilution), and ICAM1 (60299-1-Ig, Proteintech, 1:1000 dilution) overnight at 4 °C. Thereafter, the blots were washed three times and incubated with secondary antibodies for 50 min at 37 °C. Subsequently, the blots were washed three times and the signals were detected using the Pierce™ ECL Western Blotting Substrate (32,106, Thermo Fisher Scientific) and Millipore Immobilon™ Western Chemiluminescent HRP Substrate (WBKLS0100, Millipore) at 25 °C.

### Mass spectrometry identification and data analysis

The EpSC-derived extracellular vesicles were collected and digested using the protein lysis buffer. Thereafter, the protein mixtures were extracted, processed, and digested as described previously [23]. The peptide mixtures were analyzed using an Orbitrap Fusion Mass Spectrometer equipped with a nanoflow liquid chromatography system. All MS/MS raw files were processed using the MaxQuant software [24] (version 1.6.5.0), searched against the UniProt human database (https://www.uniprot.org/, downloaded on April 2020). Protein quantification was performed according to the intensity-based absolute quantification method [25] as implemented in MaxQuant. The online tool DAVID (https://david.ncifcrf.gov/) [26] was used to annotate the proteins according to biological processes, cellular components, and molecular functions. The Matrisome database [27] (http://matrisomeproject.mit.edu/) was used to annotate the extracellular matrix (ECM) proteins. Protein–protein interactions were retrieved from the online tool STRING [28] and the visualizations were built using Cytoscape [29] (version 3.7.2).

### Statistical analysis

For all results, the statistical data are shown as means ± S.E.M. Student’s *t* test was used to compare data between groups. ANOVA was used to compare three or more groups. Replicates used were biological replicates. Results were considered significant at *p* ≤ 0.05. Statistical tests were carried out using the GraphPad Prism 6 (La Jolla, CA, USA) software for Apple Mac.

## Results

### Establishment of human EpSC culture

**Fig. 1:**
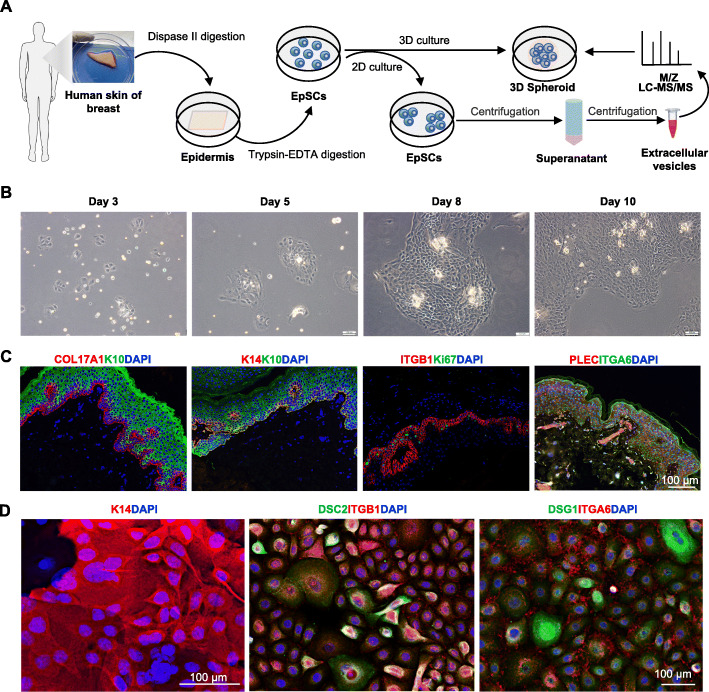
Establishment of human epidermal stem cell (EpSC) Culture. **a** Schematic overview of tissue processing, EpSC culturing, and extracellular vesicle analysis. **b** Representative images show the generation of human EpSCs (scale bar 100 μm). **c** Immunostaining of COL17A1, K14, K10, ITGB1, Ki67, PLEC, and ITGA6 in human skin (scale bar 100 μm). **d** Immunostaining of K14, DSC2, ITGB1, DSG1, and ITGA6 in EpSCs (scale bar 100 μm). All biological experiments were repeated three times (*n* = 3, biological replicates)

The epidermis was separated from human skin tissue with Dispase II digestion. Thereafter, the epidermal cells were isolated from the epidermis by trypsin digestion for the 2D culture on matrix gel (Fig. 1a). The adherent cells could grow clonally over time (Fig. 1b). EpSCs located in the basal layer of the human skin tissue [30], which expresses with stem markers (K14 and collagen type XVII alpha 1 chain, COL17A1), adhesive receptor (integrin subunit beta 1, ITGB1), and hemidesmosome complexes (integrin subunit alpha 6, ITGA6, and plectin, PLEC) (Fig. 1c) [31]. Immunofluorescence staining showed that almost all clonally cultured epidermal cells expressed K14 (Fig. 1d). The EpSCs have capacity of adhesion since the cell-matrix junction markers (ITGA6) and cell-cell junction markers (desmosome, DSC2, and DSG1) were expressed in the EpSCs (Fig. 1d), indicating that the cultured EpSCs exhibit polarity. These results indicate that a 2D culture system of human EpSCs was successfully established.

### Wnt/β-catenin signal promotes the stemness of EpSCs

**Fig. 2:**
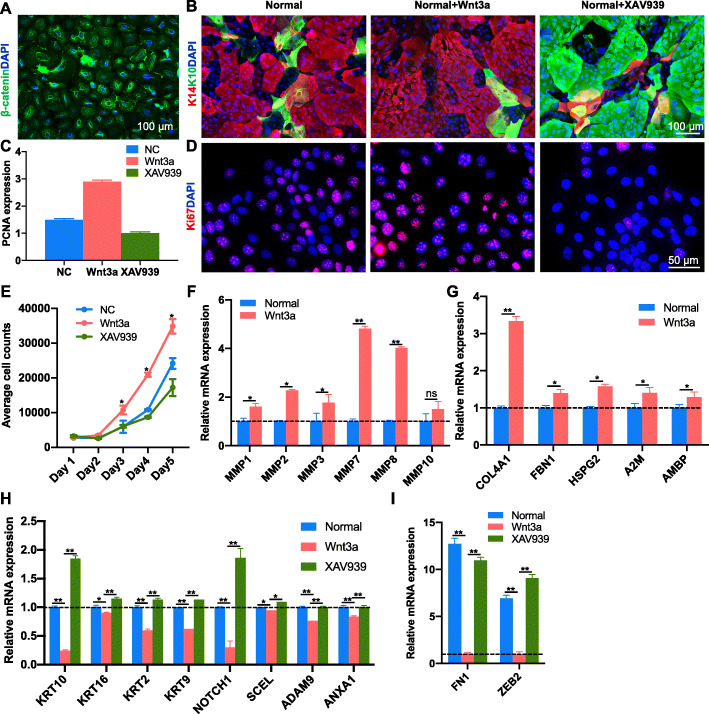
Wnt/β-catenin signaling promotes stemness and function of interfollicular epidermal stem cells (EpSCs). Immunostaining of β-catenin (**a**), K14 and K10 (**b**), and Ki67 (**d**) in EpSCs treated with Wnt3a or XAV939. **c** mRNA expression of *PCNA* in EpSCs treated with Wnt3a or XAV939. **e** Proliferation curve of EpSCs analysis in EpSCs treated with Wnt3a or XAV939. mRNA expression of MMPs (**f**), BM components (**g**), keratinocyte differentiation associated genes (**h**), and EMT associated genes (**I**), in EpSCs treated with Wnt3a or XAV939. All biological experiments were repeated three times (*n* = 3, biological replicates; data displayed as mean ± SEM, *p < 0.05, **p < 0.01)

The Wnt signaling pathway is essential for epidermal stem cell self-renewal [32]. β-catenin, the key protein of the Wnt pathway, translocated into nucleus (Fig. 2a), indicating the expansion potential of the EpSCs. The EpSCs were treated with the β-catenin inhibitor (XAV939) to study the role of the Wnt pathway on fate determination. The results showed that more mature marker proteins (K10) were present in the XAV939 group compared to the control groups. However, the stemness marker (K14) was found to be upregulated with a little K10 after Wnt3a treatment (Fig. 2b). Proliferation markers (PCNA and Ki67) and proliferation curve revealed that the proliferation ability of EpSCs was enhanced after treated by Wnt3a (Fig. 2c–e). In addition, we found that Wnt3a-treated EpSCs could express more ECM-digesting matrix metallopeptidases (MMPs), such as *MMP1*, *MMP2*, *MMP3*, *MMP7*, *MMP8*, and *MMP19*, compared to the control group (Fig. 2f). ECM digestion is a secondary effect of cell proliferation [33], indicating that the proliferation ability of Wnt3a-treated EpSCs was higher. More ECM proteins, such as *COL4A1*, fibrillin 1 (*FBN1*), heparan sulfate proteoglycan 2 (*HSPG2*), alpha-2-macroglobulin (*A2M*), and alpha-1-microglobulin/bikunin precursor (*AMBP*), of the BM, an important EpSC niche, were found in Wnt-treated EpSCs compared to the control group (Fig. 2g). On the other hand, the genes related to keratinocyte differentiation were downregulated in Wnt3a-treated EpSCs, and recovered in XAV939 group (Fig. 2h). The genes involved in epithelial mesenchymal transition (EMT), such as fibronectin 1 (*FN1*) and zinc finger E-box binding homeobox 2 (*ZEB2*), were downregulated in Wnt3a-treated EpSCs; however, they were upregulated in the XAV939 group (Fig. 2i), indicating the importance of the Wnt pathway on epidermization. These results indicate that activation of the Wnt/β-catenin signaling pathway is necessary for determining EpSC fate.

### EpSC-derived extracellular vesicles mediate EpSC development

**Fig. 3:**
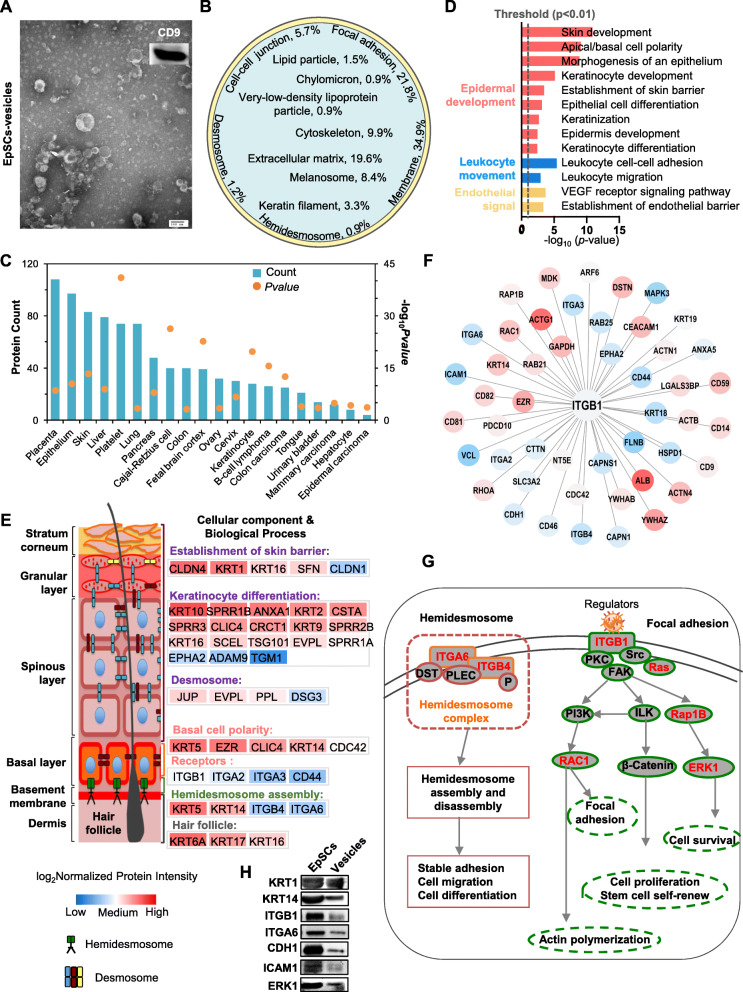
Epidermal stem cell (EpSC)-derived extracellular vesicles mediate EpSC development. **a** Transmission electron microscopy (TEM) and western blot analysis of EpSC-derived extracellular vesicles. **b** Schematic of percentage of cell components in EpSC-derived extracellular vesicles. Tissue expression (**c**) and biological process (**d**) analysis of EpSC-derived extracellular vesicles. **e** Schematic of cellular component and biological process in spatial function of the epidermis. The depth of the color indicates protein abundance according to log_2_ normalized protein intensity of EpSC-derived extracellular vesicles. **f** Interaction network between ITGB1 and other proteins of EpSC-derived extracellular vesicles. **g** Selected cellular pathways of hemidesmosome and focal adhesion. **h** Western blot analysis of KRT10, KRT14, ITGB1, ITGA6, ERK1, ICAM1, and ECAD in EpSCs and EpSC-derived vesicles. All biological experiments were repeated three times (*n* = 3, biological replicates)

Stem cell-derived extracellular vesicles are useful in regenerative medicine. To study the role of EpSC-derived extracellular vesicles in epidermal development, we collected extracellular vesicles from cultured EpSCs for proteomic analysis (Fig. 3a). Our results showed that EpSC-derived extracellular vesicles were enriched in cytoskeletal components, including the membrane (34.9%), cytoskeleton (9.9%), and keratin filament (3.3%); cell adhesion molecules, including focal adhesion (21.8%), hemidesmosome (0.9%), cell-cell junction (5.7%), and desmosome (1.2%); lipid related proteins, including lipid particles (1.5%), chylomicron (0.9%), and very-low-density lipoprotein (0.9%); melanosomes (8.4%); and extracellular matrix (19.6%) (Fig 3b, Supplementary Table S2). Tissue expression analysis showed that EpSC-derived extracellular vesicles were enriched in the epithelium and skin (Fig. 3c), indicating that most EpSC-derived extracellular vesicles may be epithelium specific and skin specific.

Biological process analysis showed that the proteins present in EpSC-derived extracellular vesicles were involved in epidermis development, including polarity, morphogenesis, and differentiation of epithelial cells, and the establishment of the skin barrier (Fig. 3d). In addition, processes of leukocyte movement and endothelial signal were also enriched in EpSC-derived extracellular vesicles. To evaluate the role of EpSC-derived extracellular vesicles in epidermal development, the functions of the EpSC-derived extracellular vesicles were analyzed according to the spatial function of skin. Our results showed that extracellular vesicle proteins were involved in functions associated with the dermal and epidermal layers of the skin, e.g., the hair follicles at the dermal papilla of the dermis (KRT6A, KRT17, and KRT16), receptors (ITGB1, ITGA2, ITGA3, and CD44) and polarity of the basal layer of epidermis (KRT5, EZR, CLIC4, KRT14, and CDC42), keratinocyte differentiation (KRT10, SPRR1B, ANXA1, KRT2, CSTA, SPRR3, CLIC4, CRCT1, KRT9, SPRR2B, KRT16, SCEL, TSG101, EVPL, SPRR1A, EPHA2, ADAM9, and TGM1), and establishment of barrier function of the epidermis (CLDN4, KRT1, KRT16, SFN, and CLDN1) (Fig. 3e). In addition, the important components of the dermis-epidermis junction (DEJ), including KRT5, KRT14, ITGB4 and ITGB6, were also enriched in EpSC-derived extracellular vesicles (Fig. 3e).

ITGB1 is important for signal transduction in EpSCs. Interaction-network analysis showed that 48 proteins of EpSC-derived extracellular vesicles were associated with ITGB1 (Fig. 3f), indicating a role in signal transduction. Some were involved in the ITGB1-FAK pathways of EpSCs including members of the RAS superfamily (RAB25, RAP1B, and RAC1) and the MAP kinase family (MAPK3/ERK1) (Fig. 3f, g). Further, the stemness marker (K14), mature marker (K1), integrins (ITGB1 and ITGA6), cadherin (CDH1), adhesion molecule (ICAM1), and ERK1 were verified expressed in the vesicles (Fig. 3h). The results enabled us to evaluate whether EpSC-derived extracellular vesicles were associated with signaling pathways by activating ITGB1. Surprisingly, 36 soluble regulators of ECM were identified in EpSC-derived extracellular vesicles according to the Matrisome database [27]. Some were involved in cell growth, such as cell proliferation (ANXA1, S100P, and FGFBP1), cell differentiation (ANXA5, S10017A, CRNN, S100A8, and MDK), and cell migration (ADAM9, S100A14, and S100A6) (Supplementary Table S3). For example, galectins (LGALS7), which are specifically expressed in the keratinocytes of the squamous epithelium, can directly function in epidermal cell growth. A protein group associated with the immune response and inflammation, such as ANXA5, S100A7A, IL1RN, IL18, and CRNN, was also identified. In addition, enzyme system related proteins, including proteases (CTSD and ADAM9) and protease inhibitors (CSTA, CSTB, SERPINB1, SERPINB2, SERPINB3, SERPINB5, and MUC20), were identified. Together, these results indicate that EpSC-derived extracellular vesicles can promote the function and development of EpSCs through signal transduction.

### Establishment of a functional human epidermal 3D organoid culture

**Fig. 4:**
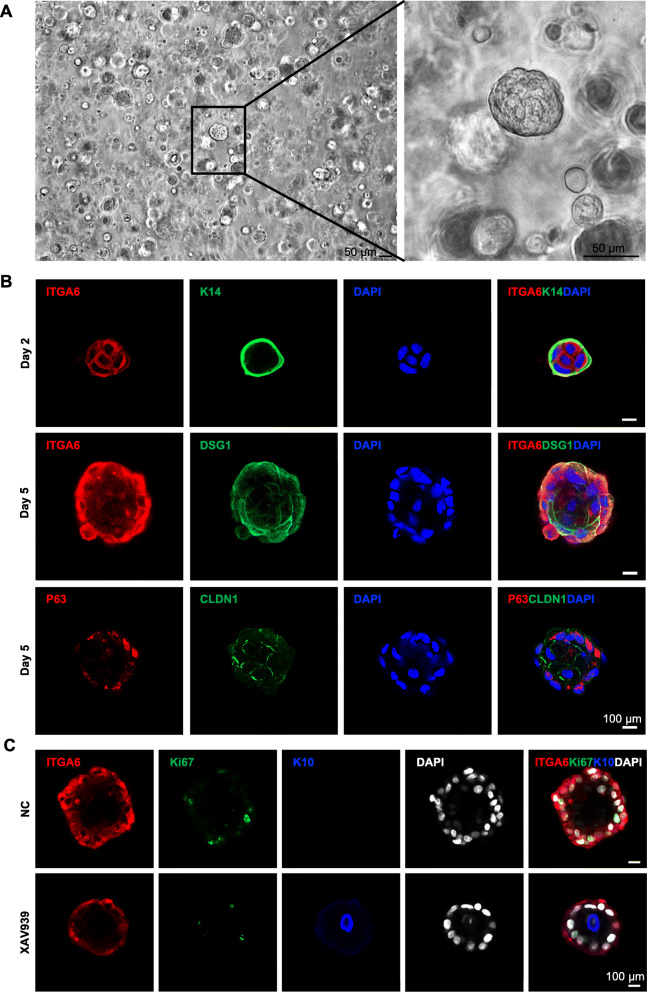
Establishment of functional human epidermal 3D organoid culture. **a** Representative images show the generation of human epidermal 3D organoids (scale bar 50 μm). **b** Immunostaining of ITGA6 and K14 at day 2 and ITGA6, DSG1, P63, and CLDN1 at day 5 (scale bar 100 μm). **c** Immunostaining of ITGA6, Ki67, and K10 at day 10 (scale bar 100 μm). All biological experiments were repeated three times (*n* = 3, biological replicates)

EpSCs derived from human tissues can mimic the cellular composition of their tissue-of-origin in 3D cultures called organoids [34]. To promote the function of 3D organoids, we designed an organoid culture system for the human epidermis using Wnt3a treatment for the first 5 days to maintain EpSC stemness (Fig. 4a). Immunofluorescence staining showed that the organoids expressed the stemness (K14, Fig. 4b) and proliferation (Ki67, Fig. 4c) markers at high levels. In addition, the transcription factor (P63) of EpSCs was expressed in the 3D culture (Fig. 4b). To retain the support of EpSC-derived extracellular vesicles on organoid function, we maintained fresh medium in a 1:1 ratio. Results showed that cell-cell junction markers (DSG1 and CLDN1) and cell-matrix junction marker (ITGA6) were expressed well. Further, XAV939 was added to promote EpSC differentiation into mature epidermal cells at day 6. Five days later, the mature marker (K10) was found in 3D organoids. These results indicate that we have successfully established a 3D culture system for EpSCs with the ability to proliferate and differentiate.

## Discussion

The interaction between stem cells and stem cell niches is a hot topic in research. The EpSC niche of skin (BM) is important for its stemness in the natural state. We found that EpSCs isolated from the human skin and cultured with Wnt3a can express more components of the BM, including COL4A1, FBN1, HEPG2, A2M, and AMBP (Fig. 2g), indicating that EpSCs can promote BM formation for tissue engineering or stem cell transplantation. Secondly, Wnt3a enhanced EpSCs’ proliferation, and various MMPs, which enhance EpSCs proliferation by degrading BM for self-renewal, were detected at a high level in Wnt3a-treated EpSCs. Thirdly, proteins (FN1 and ZEB2) associated with EMT decreased after Wnt3a treatment and increased after treatment with the inhibitor of β-catenin (XAV939) in EpSCs. EMT has been reported existing in stem cells, differentiated cells, and involved in many important physiological and pathological processes [35, 36]. For example, skin injury needs re-epithelialization, which is considered as a partial EMT process [37], by the migration and proliferation of epidermal basal stem cells during wound healing [38]. In addition, the loss of epithelial polarity of epidermal cells in the EMT affects epidermal development and morphogenesis [39, 40]. Therefore, EMT could be an important factor to consider for epithelium characterization. These results indicated that the Wnt/β-catenin pathway was important for EpSC expansion and function. 3D organoids reflect near-physiological cellular composition and behavior than traditional 2D cultures [41]. In this study, we used Wnt3a protein, which is important for the self-renewal of EpSCs, as an additive to cultivate 3D organoids. Wnt3a was added in the early stages to improve the stemness and specific function of EpSCs in 3D culture.

The proteomic and functional characterization of EpSC-derived extracellular vesicles was also mapped. We found that EpSC-derived extracellular vesicles participate in the development of EpSCs, indicating the autocrine role of EpSCs. EpSC-derived extracellular vesicles were involved in each stage of development—from basal to mature layer—of interfollicular epidermal stem cells, including the hair follicles of the dermis, the EpSCs of the epidermal layer and the EpSC microenvironment, and the terminally differentiated epidermal layer. The epidermal development of the skin is related to the hemidesmosomes and focal adhesion. Hemidesmosomes are associated with the proliferation, migration, and differentiation of EpSCs through dynamic changes in assembly and disassembly to maintain homeostasis and wound repair. ITGB1 is a focal adhesion complex and regulates EpSC development and function through signal transduction. Our results indicated that the expression of hemidesmosome complexes and ITGB1 could be regulated by the form of EpSC-derived extracellular vesicles. In addition, 36 known ECM soluble factors were found to be secreted in the form of extracellular vesicles, possibly activating ITGB1 as growth factors. Many were directly involved in the maintenance and function of epidermal cells, further indicating the role of EpSC-derived extracellular vesicles in the functional culture of EpSCs. Finally, using the fluid secreted by EpSCs and the Wnt/β-catenin inhibitor (XAV939), we obtained 3D organoids which were close to the epidermis, including basal and mature layers after 10 days.

## Conclusion

In this study, we demonstrated the role of the Wnt pathway in enhancing the EpSCs’ function through 2D/3D systems, as well as the potential role of EpSC-derived extracellular vesicles on skin development. These results provide novel directions for the improvement of skin organoids and tissue-engineered skin and stem cells for clinical transplantation and drug screening.

## Supplementary information

**Additional file 1: Supplementary Table S1.** Details of primer sequences used for PCR in this study.

**Additional file 2: Supplementary Table S2.** Proteome profile of epidermal stem cell (EpSC)-derived extracellular vesicles.

**Additional file 3: Supplementary Table S3.** List of extracellular matrix (ECM) proteins identified in epidermal stem cell (EpSC)-derived extracellular vesicles.

## Data Availability

The proteomics dataset supporting the conclusions of this article is available in the ProteomeXchange Consortium via the iProX [42] with the dataset identifier PXD020351 (http://proteomecentral.proteomexchange.org/cgi/GetDataset?ID=PXD020351).

## Abbreviations

BM: Basement membrane
DEJ: Dermis-epidermis junction
ECM: Extracellular matrix
EMT: Epithelial mesenchymal transition
EpSC: Epidermal stem cell
TEM: Transmission electron microscopy
